# TRPM2-mediated Ca^2+^ signaling as a potential therapeutic target in cancer treatment: an updated review of its role in survival and proliferation of cancer cells

**DOI:** 10.1186/s12964-023-01149-6

**Published:** 2023-06-19

**Authors:** Eunus S. Ali, Brototi Chakrabarty, Sarker Ramproshad, Banani Mondal, Neloy Kundu, Chandan Sarkar, Javad Sharifi-Rad, Daniela Calina, William C. Cho

**Affiliations:** 1 College of Medicine and Public Health, Flinders University, Bedford Park, 5042 Australia; 2 Gaco Pharmaceuticals, Dhaka, 1000 Bangladesh; 3 Present Address: Department of Biochemistry and Molecular Genetics, and Simpson Querrey Institute for Epigenetics, Northwestern University Feinberg School of Medicine, 303 E Superior St, Chicago, IL 60611 USA; 4 Pharmacy Discipline, Khulna University, Khulna, 9208 Bangladesh; 5 Department of Pharmacy, Ranada Prasad Shaha University, Narayanganj, 1400 Bangladesh; 6 Department of Pharmacy, Bangabandhu Sheikh Mujibur Rahman Science and Technology University, Gopalganj, 8100 Bangladesh; 7 Facultad de Medicina, Universidad del Azuay, Cuenca, Ecuador; 8 Department of Clinical Pharmacy, University of Medicine and Pharmacy of Craiova, Craiova, 200349 Romania; 9 Department of Clinical Oncology, Queen Elizabeth Hospital, Kowloon, Hong Kong, China

**Keywords:** TRPM2 ion channel, Ca^2+^ signaling, Oxidative stress, Cancer, Therapeutic target

## Abstract

The transient receptor potential melastatin subfamily member 2 (TRPM2), a thermo and reactive oxygen species (ROS) sensitive Ca^2+^-permeable cation channel has a vital role in surviving the cell as well as defending the adaptability of various cell groups during and after oxidative stress. It shows higher expression in several cancers involving breast, pancreatic, prostate, melanoma, leukemia, and neuroblastoma, indicating it raises the survivability of cancerous cells. In various cancers including gastric cancers, and neuroblastoma, TRPM2 is known to conserve viability, and several underlying mechanisms of action have been proposed. Transcription factors are thought to activate TRPM2 channels, which is essential for cell proliferation and survival. In normal physiological conditions with an optimal expression of TRPM2, mitochondrial ROS is produced in optimal amounts while regulation of antioxidant expression is carried on. Depletion of TRPM2 overexpression or activity has been shown to improve ischemia–reperfusion injury in organ levels, reduce tumor growth and/or viability of various malignant cancers like breast, gastric, pancreatic, prostate, head and neck cancers, melanoma, neuroblastoma, T-cell and acute myelogenous leukemia. This updated and comprehensive review also analyzes the mechanisms by which TRPM2-mediated Ca^2+^ signaling can regulate the growth and survival of different types of cancer cells. Based on the discussion of the available data, it can be concluded that TRPM2 may be a unique therapeutic target in the treatment of several types of cancer.

Video Abstract

**Supplementary Information:**

The online version contains supplementary material available at 10.1186/s12964-023-01149-6.

## Introduction

The transient receptor potential melastatin 2 (TRPM2) channel, under the transient receptor potential (TRP) ion channel superfamily, is engaged in a range of physiological and pathophysiological processes in a variety of cells. Genomic study of *Drosophila* first recognized the *trp* gene through visual transduction mutation [1, 2]. TRPM2, a decade’s sought-after ion channel, also formerly called TRPC7 and LTRPC-2, a nucleotide-sensing TRP channel enables the influx of Ca^2+^ and Na^+^ and increases the cytosolic Ca^2+^ concentration, [Ca^2+^]_cyt_. It also works as a lysosomal Ca^2+^ release channel [3] involving, under certain situations, cell death. TRPM2 channel is expressed in liver cells [4], blood cells, immunocytes, pancreatic cells, microglia [5–7] and brain cells [8]. In addition, extracellular application of oxidants e.g., H_2_O_2_ [9–12], tert-butyl hydroperoxide and dithionite, second messenger arachidonic acid [10], intracellular ADP-ribose (ADPR) [11], and oxidative stress e.g., H_2_O_2_ [9–12], nicotinamide adenine dinucleotide (NAD) [6] can act as an activator of TRPM2 channel gating. The physiological role of TRPM2 is very less understood. TRPM2 channels may be involved in insulin secretion [3, 10, 13–17]; it also may facilitate portions of the responses to tumor necrosis factor alpha (TNF-α), in the cells of the immune system [18], cell motility, cell death [19]. Miller [20] showed that it is related to the amyloid-beta protein toxicity, in the brain. However, the molecular basis of the contribution of the TRPM2 channel to these cellular and pathophysiological processes remains unclear. As the TRPM2 channel undergoes ROS-mediated activation, and the oxidation process is involved in a variety of diseases and clinical problems, the TRPM2 channel may be a possible therapeutic target for those disease states. This review aims to focus on recent findings in TRPM2 Ca^2+^ permeable channel in IR injury and cancers, and its activation mechanism by ROS in different cell types and its associated implications.

## Review methodology

This updated review analyzed the pharmacological studies that included the mechanisms of how oxidative stress modulates TRPM2, Ca2+ signaling mediated by TRPM2, regulation mechanisms in ischemia and in various types of cancer. For this purpose, searches were performed in specialized databases such as Web of Science, Pubmed/MedLine, ScienceDirect, and TRIP Database using the following MeSH terms: “Humans”, “Oxidative Stress”, “TRPM Cation Channels/physiology”, “Calcium/metabolism”, “Cell Survival”, “Humans”, “Molecular Targeted Therapy”,”Oxidative Stress”, “TRPM Cation Channels/genetics”, “TRPM Cation Channels/metabolism”, “Transcriptional Activation”, “Neoplasms/genetics”. The most relevant data were summarized in a table and a figure.

## RPM2 channel: a brief overview

### Structure and properties

The pharmacology and structural studies of TRPM2 remain less developed. It belongs to the TRPM subfamily, which takes part of the TRPM-homology section (around 700 amino acids) within the N-terminus. The human TRPM2 gene, situated in chromosome 21q22.3, comprises 32 exons and spans about 90 kb [21]. The TRP protein has six putative transmembranes forming the sensor domain by helices S1-S4 [22] along with a pore area within the fifth and sixth transmembrane; besides it gathers in homo- or hetero-tetramers form channels [23]. As it undergoes tetramerization, the putative S5 and S6 segments of the TRPM2 channel can form a central ion-conducting pore. The bacterial KcsA potassium channel has a similar type of ion channel pore structure [24]. The C-terminal end of TRPM2 has a 39% sequence similarity to NUDT9 (Nudix (nucleoside diphosphate linked moiety X-type motif 9). Between the NUDT9 domain (comprising some 300 amino acids) and the Nudix box is the catalytic domain containing 22 amino acids. Eisfeld and Luckhoff [25] suggest that the NUDT9 domain, probably, may provide long-term binding of ADPR which can be important for channel gating. Kuhn and Luckhoff [26] reported that one particular amino acid (Asn-1326) can play a critical role in the binding area for ADPR (adenosine diphosphate (ADP)–ribose) gating of TRPM2. In addition, cytoplasmic N-terminally located TRPM homology regions may be involved for the oligomerization of channels or in regulating transport to the plasma membrane [22]. Although TRPM2 and TRPM8 channels are structurally 42% alike [27], they are highly different in their biological activities. The single-channel characteristics of TRPM2 are distinctive as the channel shows extensive opening times [28, 29]. Like TRPM6/7, TRPM2 is also familiar as a ‘coenzyme’ due to its double actions in the ion channel as well as the C-terminal enzyme domain [30, 31]. However, the proteolytic action of TRPM2’s Nudix box assumes to be tremendously small or the role of the TRPM2 channel is abolished [25].

### Physiological functions of TRPM2

The physiological role of TRPM2, although it is expressed in various tissues and first identified in 1998, is not very well understood. However, some propositions are available regarding the physiological function of the TRPM2 channel. It might be involved in insulin secretion [13]; it may arbitrate parts of the reactions to TNF-α, within immune cells [32]. In addition, H_2_O_2_-induced TRPM2 channels contribute to alloxan-induced diabetes mellitus [33]. It is also proposed that within the brain it may cause toxicity of amyloid-beta which is a protein related to Alzheimer’s disease [20]. In addition, the channel may be also involved in cell motility and cell death [19]. A recent study revealed that mice having TRPM2- deficiency were tremendously prone to infection with *Listeria monocytogenes* (Lm), showing an ineffective intrinsic immune response. Therefore, to survive and control the bacterial burden of Lm infection, TRPM2 may play a crucial role [34].

**Fig. 1:**
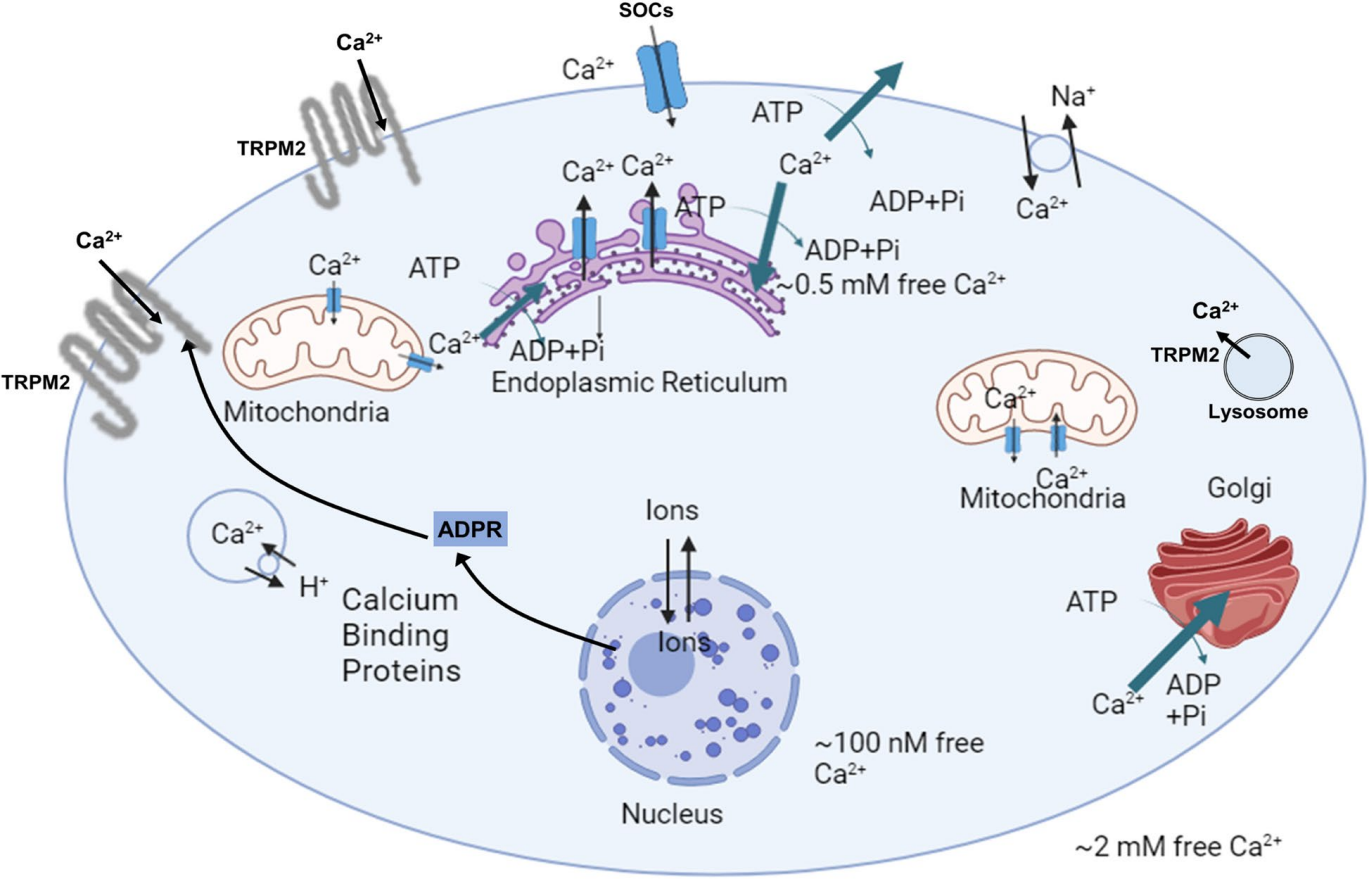
Schematic representation of cellular calcium homeostasis and cellular location of TRPM2 channels. Abbreviations: Transient receptor potential melastatin 2 (TRPM2), Adenosine Diphosphate (ADP), phosphates (Pi), Adenosine triphosphate (ATP)

TRPM2 is one of several Ca^2+^ entry channels in mammalian cells. Ca^2+^ plays a very essential role as an intracellular messenger. It is a second messenger and an important regulator of cellular metabolism [35–39] (Fig. 1). Cytoplasmic Ca^2+^ regulates certain key cellular systems. More than one hundred neurotransmitters and hormones, which work on their corresponding receptors and receptor channels transmit their signals by changing intracellular calcium concentration. In cells, Ca^2+^ does work from minutes to hours to guide gene transcription and cell proliferation. Ca^2+^ passes via TRPM2 triggered by ROS and may prompt chemokine assembly in monocytes which consequently may exacerbate inflammatory neutrophil penetration [40]. In cancers or other disease conditions, the expression of TRPM2 ion channels along with others is significantly altered causing impaired intracellular calcium homeostasis. While normal intracellular calcium concentration/homeostasis is essential for cellular metabolisms, altered calcium homeostasis may play a critical role in the development and progression of many detrimental diseases [41]. The plasma membrane of mammalian cells possesses different types of Ca^2+^ entry channels that control the downhill diffusion of Ca^2+^ entry into cells [42].

Types of calcium channels according to their gating mechanisms:

1. Voltage-dependent like N, L, T, P, and Q types of calcium channels;
2. Mechanically-gate activated like Stretch activated, non-selective calcium channels;
3. Ligand-gated activated like:
  i) External ligands such as neurotransmitters (nicotine acetylcholine receptor)
  ii) Intracellular ligands such as IP3, Ca^2+^, ATP (IP_3_ receptor or store-operated channels [43].

After depletion of Ca^2+^from the key store of Ca^2+^ (endoplasmic reticulum) of mammalian cells, a special plasma membrane Ca^2+^ channel, the CRAC channel is triggered to adjust gradually the extent of calcium in the endoplasmic reticulum. CRAC (Ca^2+^ release activated Ca^2+^) channel are the best characterized SOCE channels (store-operated Ca^2+^ entry channels) with well-studied electrophysiological properties [44]. The putative location of TRPM2 has been indicated in Fig. 1.

### Mechanism of activation of TRPM2 channel

#### Activation mechanism of TRPM2 channel by oxidative stress

Oxidative stress is generally mediated by extreme disclosure of cells to reactive oxygen or nitrogen species, generated by the ischemic attack, radioactivity, seizure, shock, etc. Experience with oxidative stress prompts apoptotic-like late death of neurons as well as decay in cell culturing, facilitated through increased Ca^2+^, malfunction of mitochondria as well as stimulation of poly[adenosine diphosphate ribose (ADPR)] polymerase (PARP) because of DNA injury [45, 46]. On this matter, one possibly vital calcium influx way can be the stimulation of Na^+^ and Ca^2+^ penetrable TRP channels. The cell damage produced by ischemia followed by reperfusion is mainly for superoxide anions [47]. These superoxide anions will produce H_2_O_2_ which helps further produce ADPR in the nucleus. H_2_O_2_ and superoxide anions are potential members of ROS [10]. ROS, one class of molecules and ions has the potential for the destruction of cells. They have extremely reactive features and are, generally, produced from aerobic metabolism through the mitochondrial electron transport chain resulting from exposure to extracellular mediators and cases, like ionizing emission, cytotoxic medicines, ischemia–reperfusion injury, and hypoxia-reoxygenation [48–50]. Nicotinamide adenine dinucleotide (NAD^+^) can support the TRPM2 opening over ROS stimulus [51]. Presumably, the transformation from NAD^+^ to ADP-ribose can trigger this opening [11, 52]. But surprisingly, NAD was not active in TRPM2 transduced HEK-293 cells [53]. It has been suggested that, in the case of cardiomyocytes, stimulation of the TRPM2 channel, as well as poly(ADP-ribose) polymerase, is expected to be engaged in oxidative stress-prompted cell death [54]. In cancer, the production of ROS by mitochondria promotes cellular oncogenesis by shaping signaling pathways and specific transcription factors [55]. TRPM2 maintains the bioenergy of cancer cells by maintaining mitochondrial activity and increased ROS production [38, 56]. TRPM2 inhibition decreases ROS production by modulating Nrf2, decreasing NADH and NADPH production, thus causing cancer cell death [57]. Recent studies have also shown that TRPM2 preserves cell viability in the case of other non-tumor cells, protecting them from ischemia–reperfusion injury [58, 59]. According to recent studies it is evident that TRPM2 plays a fundamental role for cardiac myocyte bioenergetics, limits oxidative stress, preserves mitochondrial function and protects the heart (as well as other tissues) from I/R insult. On the other hand, acting through similar mechanisms, TRPM2 is an important player for reducing tumour growth and the survival of cancer cells, also protecting cells from the toxicity of some anticancer drugs (i.e. doxorubicin) [60]. Therefore, by reducing mitochondrial dysfunctions, decreasing free radicals production, TRPM2 channels can protect cardiomyocytes and the heart from I/R injuries [59]. Glutamine consumption is substantially increased in many types of cancer compared to other amino acids, representing a characteristic of malignancy. Glutaminolysis is an alternative energy source for cancer cells and it is a key nutrient for several metabolic processes leading to: ATP generation, redox homeostasis, intracellular antioxidant stock maintenance, and macromolecular synthesis. Regulation of glutaminolysis is determined by oncogenic signals [61]. Recent studies have shown that TRPM2 inhibition decreases glutamine production, thus decreasing antioxidant cofactors and the antioxidant response, increasing cancer cell death and reducing tumor growth [7, 57].

In neuronal cells, TRPM2 channels can be associated with neuronal destruction triggered by means of oxidants, amyloid β-peptide along with tumor necrosis element alpha. Activation of the TRPM2 channel can be influenced by different types of endogenous factors or proteins. The opening of the TRPM2 channel in response to oxidative stress may depend upon the stimulation of DNA restoration enzyme poly (ADP-ribose) polymerase [12]. They also hypothesized that PARP enzyme action may be a crucial element of the pathway relating to oxidative stress with TRPM2 instigation. Zhang, Tong et al. [62] reported that TRPM2 can be quickly tyrosine phosphorylated after the stimulation with H_2_O_2_ or TNF-α and phosphorylation is of paramount importance for the activation of the channel; the protein tyrosine phosphatase – L1 (PTPL1) also is associated with the modulation of TRPM2 activation. TRPM2 can be a target for dephosphorylation and inactivation by PTPL1. The molecular mechanism that may influence tyrosine phosphorylation of TRPM2 is not clearly understood yet. Presumably, the pathway may include phosphorylation of tyrosine in the NUDT9-H domain of TRPM2, phosphorylation of calmodulin-binding sites, or phosphorylation of sites that may influence the tertiary structure of TRPM2. Superoxide anions and H_2_O_2_ may trigger cellular death through various mechanisms. H_2_O_2_, probably, does not activate MAPK signaling and superoxide anions-induced apoptosis is dependent on JNK activity. H_2_O_2_ and/or other ROS can release Ca^2+^ from the intracellular Ca^2+^ pools and activate suppressors of cytokine signaling (SOCs). Also, during oxidative stress, H_2_O_2_ may enter the cytosol by an unidentified mechanism. Using the Fenton reaction, altogether H_2_O_2_ and Fe^3+^ can generate hydroxyl radical (OH–) within the cytosol of cells. TRPM2, also, maybe straightly stimulated by H_2_O_2_ and OH– radicals [63]. Interestingly, in HEK293/hTRPM2 cells, the TRPM2 channels have been revealed to be engaged in H_2_O_2_- activated cell death in Ca^2+^ independent mechanism [64].

Attachment of ligand to G protein-linked receptors might result in ADPR production. Receptor stimulation also increases within the intracellular Ca^2+^ concentration through IP_3_- mediated Ca^2+^ transportation from stores. The arrival of Ca^2+^ into the cell by TRPM2 delivers a positive response augmentation of TRPM2 stimulation. In a similar mechanism connected to that followed by H_2_O_2_, the pro-diabetic medication alloxan, [25] leads to TRPM2 triggering. Moreover, the nitric oxide synthase (NOS) enzyme is stimulated by diacylglycerol (DAG) through arachidonic acid. Nitric oxide (NO) radicals can be derived from L-arginine through NOS. Furthermore, NO may trigger TRPM2 channels [63, 65]. By applying the activation mechanism, TRPM2 can be a potential candidate for gene therapy. For a detailed activation mechanism of TRPM2 during oxidative stress, readers are requested to read through some of the recent reviews indicated here [66–68]. It has been reported that in glioblastoma cells, cell death by Ca^2+^ elevation after H_2_O_2_ usage could be facilitated by the insertion of TRPM2 into A172 cells [69].

#### ADPR role in regulation of the TRPM2 channel

The inhibitory function of a small unite variant of TRPM2 on its extended, pore-creating isoform has been stated for TRPM2 [70, 71]. An important regulatory mechanism has been reported through the particular stimulation of TRPM2 channels by intracellular ADP-ribose (ADPR). It has been recommended to be the prime gating mechanism of TRPM2 [71, 72]. TRPM2 channel action is controlled by various cytosolic aspects, involving cyclic ADPR (cADPR), nicotinamide adenine dinucleotide phosphate (NAADP), Ca^2+^, calmodulin (CaM) as well as adenosine monophosphate (AMP) [73]. In addition, currents of TRPM2 are disabled when intracellular Ca^2+^ content decreases under 100 nM compared to extracellular Ca^2+^ content [73]. TRPM2 is triggered by the formation of ADPR in response to oxidative stress, which attaches to the C-terminus of TRPM2, leading to open the channel [74]. ADP-ribose polymers, the source of ADPR, are combined by poly-ADP–ribose polymerase (PARP) along with hydrolyzed through poly-ADP–ribose glycohydrolase. The breaking of NAD into nicotinamide and ADP-ribose may be catalyzed by PARP enzymes after binding to oxidatively-impaired DNA [75]. PARP-reliant pathway leading to TRPM2 stimulation has been established by the usage of PARP inhibitors and these were capable to curb the H_2_O_2_-induced TRPM2 triggering [12]. In addition to that nuclear pathway, ROS can stimulate a mitochondrial pathway which consequences the assembly of ADP–ribose in mitochondria as well as in the relief of ADP–ribose to the cytosol, wherever it plays as a second messenger contributing to TRPM2 gating [52]. After gating by ADP-ribose, Ca^2+^ enters into the cells via TRPM2 which may have effective response augmentation of TRPM2 stimulation. Additionally, the triggering of TRPM2 through ADP-ribose is accelerated at elevated [Ca^2+^]_i_ indicating a positive feedback regulation of TRPM2 [76]. Other recent studies suggest that activation of TRPM2 may cause cell damage and it is expected to be engaged in various signaling pathways which may result in cell death responding to oxidative stress. Cytosolic Ca^2+^ enhances TRPM2 gating or opening by ADPR. ADPR, probably the most potent physiological activator of TRPM2, and Ca^2+^ in a concerted way may act as an important messenger system mediating Ca^2+^ influx [77, 78]. After attaching the intracellular ADPR to the NUDT9-H domain at the C-terminal, the TRPM2 channel may be activated [72]. It has been reported that endogenous ADPR concentrations in leukocytes are substantially high for activating TRPM2 when increased intracellular Ca^2+^ concentration, [Ca^2+^]_i_ is present, but perhaps not in resting [Ca^2+^] [25].

#### Effect of hypoxia/anoxia and reoxygenation on TRPM2 channel

Several researchers proposed that oxidative stress is increased by revealing cells or tissues to hypoxia. Anoxic incubation of rat liver mitochondria showed decreased free [Ca^2+^] in the mitochondrial matrix, probably, implying that during anoxia Ca^2+^ may be released into the cytoplasm of the cells. The mitochondrial respiratory chains produce most ROS in cells [79]. The level of electron movement by respiratory chain complexes controls significantly the Mitochondrial ROS manufacturing. Currently, it has been proved that in hypoxic situations, the mitochondrial respiratory chain can produce nitric oxide (NO), which can give rise to additional reactive nitrogen species [80]. In acute insults e.g., hypoxic ischemia, it has been recommended that the subsequent cell fate together with necrosis, apoptosis, or survival rely on an intracellular “Ca^2+^ setpoint” [81]. In hypoxia, TRPM2 and TRPM7 altogether have a crucial function in neuronic cell death [82] proposing that Ca^2+^ entered into cells through TRPM2 or TRPM7 caused the toxicity; TRPM2 was modulated by intracellular free radicals and conducted toxic levels of Ca^2^+ [47].

## TRPM2 in ischemic reperfusion injury

Ischemic reperfusion mainly occurs in the organ when normal blood flow in that organ suddenly or slowly stops, and after some periods, blood flow is restored. Temporary interruption of blood flow causing organ ischemia is a common occurrence during various surgical procedures. Ischemic stroke is caused by interruption of cerebral blood flow due to an obstruction and the main risk factors for ischemic, hemorrhagic and transient stroke are due to the sedentary lifestyle that generates overweight, the chronic consumption of alcohol or narcotics (cocaine, methamphetamines). Pathologies that favor the occurrence of transient ischemic, ischemic or hemorrhagic accidents are represented by heart diseases (heart failure, congenital heart malformations, heart infections, heart rhythm disorders), obstructive sleep apnea syndrome [83]. Recent studies showed the involvement of TRPM2 in pathogenesis factors of ischemic stroke as follows: by reducing oxidative stress in cardiomyocytes, TRPM2 reduces inflammation and stimulates the remodeling of the atria; it models endothelial dysfunction by deregulating the influx of calcium ions, mediates the damaging effects of ROS on the endothelium and favors hypertension; induces the death of pancreatic β cells that secrete insulin, thus promoting the development of diabetes; promotes the aggregation of platelets and favors the appearance of vascular thrombus [84–86]. In the case of hepatic ischemia–reperfusion (IR) injury, it may be considered a clinically important pathological disorder complicating liver surgery and transplantation. IR can be classified as warm IR injury as well as cold storing reperfusion injury. The hepatic surgical procedure, liver replacement, or certain kinds of liver injury can have clinical relevance with warm IR. Besides, at the time of organ preservation, cold storing reperfusion injury can occur, before liver transplantation [87]. It has been considered that long periods of ischemia may cause a drop in intracellular ATP levels, slow down active transport, and cause membrane depolarization [88]. After re-establishing the flow of oxygen and blood, reperfusion increases the damage induced by ischemia [89, 90]. Cellular cytoplasmic and mitochondrial Ca^2+^ concentrations have shown to be raised instantly following the onset of reperfusion [91]. Elevated stages of cytoplasmic Ca^2+^ have involvement in cell injury as well as death by triggering a series of Ca^2+^-dependent enzymes, also involving various proteases and phospholipases. Concurrently mitochondrial Ca^2+^ overfilling elicits a change in CsA-sensitive mitochondrial permeability transition pores (PTP) causing mitochondrial dysfunction and helping the formation of apoptosomes. In the liver, naturally occurring acidosis has been shown to delay the onset of necrotic cell death; but reperfusion normalizes the intracellular pH and the defense of acidosis vanishes. It has been suggested that intracellular acidosis also inhibits TRPM2 (pKa 6.7) [92]. In some cancerous cell lines, some evidence also suggests that altered expression of TRPM2, including some other TRP proteins, may have roles in the progression of metastatic liver cancers and hepatocellular carcinoma [43]. Overall, this information provides sufficient evidence that intracellular Ca^2+^ and TRPM2 may play a significant role in pathological conditions of the liver and other cell types. Furthermore, pharmacological inhibitors or genetically depletion of TRPM2 channels demonstrated significant protective effects in kidney I/R injury [93], cardiac I/R injury [58], and neuronal I/R injury [94].

## TRPM2 in different types of cancer

### TRPM2 in neuroblastoma

Neuroblastoma is an embryonic tumor derived from the sympathetic nervous system that occurs at the level of special nerve cells called neuroblasts [95]. Normally, these immature nerve cells transform into functional mature cells. In the case of neuroblastoma, the neuroblasts do not mature normally, but turn into cancer cells [96, 97]. TRPM2 has been revealed to cause neuroblastoma proliferation which is a non-CNS tumor of childhood as well as chemotherapy sensitivity. The leading negative small splice variant TRPM2-S first inhibited TRPM2 in expression in the cells of neuroblastoma. The inhibited TRPM2 concluded a considerably amplified vulnerability to death of the cell, prompted through low content (50–100 μM) of H_2_O_2_ [98] as well as doxorubicin [38, 71]. Mouse xenografts, using human neuroblastoma cells with expression of TRPM2-L or TRPM2-S proved the capability of TRPM2 to augment the progression of neuroblastoma tumors [71]. In neuroblastoma cells where TRPM2 was depleted, tumor development in xenografts considerably declined as well as the sensitivity of doxorubicin increased.

### TRPM2 in triple-negative breast cancer

Triple-negative breast cancer is extremely threatening, having the worst consequence, among the three main molecular types of breast cancer. However, therapy also is unsuccessful in a considerable proportion of patients suffering from estrogen-receptor-positive breast cancer, the most common class of breast cancer. 2-aminoethoxydiphenyl borate (2-APB) is known to act as a general inhibitor of several plasma membranes along with organellar ion channels involving TRPM2 [99]. TRPM2 displayed a defensive action in human breast adenocarcinoma cell lines to minimize DNA destruction, where cell proliferation declined and DNA damage climbed up significantly through pharmacological cessation of TRPM2 including 2-APB or TRPM2 mRNA silencing [100]. In the case of both triple-negative as well as estrogen-receptor-positive breast cancer, the inhibition of TRPM2 caused the rise of DNA destruction and cytotoxicity, like neuroblastoma [101]. TRPM2 was situated within the nucleus of breast adenocarcinoma but not limited to that place; about 40–45% of TRPM2 was in the nucleus while the remaining part was in subcellular sections involving the cytoplasm. The mechanisms of action of TRPM2 within the nucleus were assumed to facilitate DNA restoration through nuclear TRPM2 or elevation of the influx of nuclear calcium which was required to be investigated again. ROS content was not observed; however, the elevated oxidative stress content noticed within TRPM2-depleted neuroblastoma cells recommends the possibility to be a probable mechanism for an explanation of elevated DNA damage in breast cancer after the inhibition of TRPM2. On the other hand, within noncancerous breast epithelial cells (MCF-10A), TRPM2 was not located in the nucleus or TRPM2 blockage was detected to have a role in proliferation. These findings recommend that pointing of TRPM2 might be a synergetic method to improve the medical care of chemotherapy-resistant patients with breast cancer, resembling that proposed in neuroblastoma. Other TRPM channels are known to have a function in the case of breast cancer multiplying, movement, and invasion involving TRPM7 and TRPM8 [38, 102]. In what way do TRPM channels interpose their distinct properties and also whether their actions overlay or combine or TRPM2 are regions for imminent investigation.

### TRPM2 in lung cancer

Lung cancer results from the uncontrolled growth of abnormal cells in the lungs that do not perform the function of normal lung cells. TRPM2 is highly expressed in lung cancer [38, 103]. In non-small cell lung cancer (NSCLC), TRPM2-AS a long non-coding RNA, antisense transcript of TRPM2 was observed to be overexpressed. Besides, greater expression levels are connected with higher tumor size, progressive TNM stage, as well as reduced patient survival [104]. Cell multiplication and amplified apoptosis were considerably lowered after silencing of TRPM2-AS with siRNA (small interfering RNA). The advanced investigation will be essential to recognize the function of lncRNAs (long non-coding RNAs) including TRPM2 in cell multiplication and survival of patients as well as the consequence of expression and role of TRPM2.

### TRPM2 in digestive cancers

Oral malignant tumor or oral cancer appears as a lesion on the oral mucosa, and is caused by the division and chaotic development of cells; it can develop in any oro-maxillo-facial area, but it appears most often in the area of the tongue and floor of the mouth. In human tongue carcinoma specimens and cell lines, TRPM2 expression has been reported to be increased [105]. Treatment through 0.5 or 1 mM H_2_O_2_ amplified apoptosis in SCC9 cells of tongue carcinoma. Moreover, the breakdown of TRPM2 with siRNA augmented apoptosis, lowered survival, and hindered the movement of SCC9 cells. The subcellular location of TRPM2 was not similar in cancerous and non-cancerous cells as a considerable extent of TRPM2 protein is located in the cancer cell’s nucleus. Although in TRPM2 KO cells the processes of cell death were not discovered fully, it was not dependent on the p53-p21 pathway. The outcome is that TRPM2 has a role in the survival and movement of SCC cancer cells along with in head and neck cancers, it can be a possible therapeutic object [38, 105].

Gastric cancer begins when cancer cells form in the inner lining of the stomach. These cancer cells can grow into a tumor, and cancer usually develops slowly over several years. TRPM2 expression within tumors has a negative correlation with the overall survival of patients suffering from gastric cancer. After down-regulation of TRPM2 with shRNA in AGS and MKN-45, two gastric cancer cell lines, the cells developed slowly, and the proportion of apoptotic cells raised [38, 105]. Mitochondrial role expressed through oxygen intake amounts and ATP assembly was considerably declined in TRPM2 diminished cells as well as COX 4.1 and 4.2 expression and BNIP3 were lowered, which was described in neuroblastoma [71]. Autophagy was also declined, with a lowered amount of autophagy related genes (ATGs) involving ATG3, ATG5, ATG6, ATG7, and ATG12 and reduced transformation of LC3-I to LC3-II. Decreased autophagy led to the gathering of impaired mitochondria, lowered cellular bioenergetics, and augmented ROS, causing death of the cell. TRPM2 controlled autophagy by an mTOR (mammalian target of rapamycin) independently but JNK (Jun N-terminal Kinase) signaling reliant pathway, facilitated through regulation of ATGs, BNIP3 as well as JNK stimulation. Apoptotic properties of together paclitaxel and doxorubicin were higher in TRPM2 exhausted cells, indicating that TRPM2 conserves the survival of cells where inhibition raises sensitivities of chemotherapy and proposing this as a therapeutic attitude to increase the cell death of gastric tumors.

TRPM2 expression has a considerable role in gastric cancer cells’ bioenergetics and survival, according to confirmation from current numerous investigations [38, 66, 106, 107]. Using molecular and functional assays, one study has demonstrated that downregulated TRPM2 significantly prevents the movement and invasion capacities of gastric cancer cells, including a significant decline in the expression of metastatic markers. Besides, reduced Akt (protein kinase B) and augmented PTEN (phosphatase tensin homologue) actions were connected with the consequences. Moreover, silencing of TRPM2 concluded the deregulated metastatic markers and lost the tumor growing capacity of AGS gastric cancer cells within NOD/SCID mice. Altogether, the known outcomes offer convincing information on the vital role of TRPM2 to modulate the gastric cancer cell invasion presumably by monitoring the PTEN/Akt pathway [108]. In a recent study, Almasi et al. demonstrated that the expression of TRPM2 might be associated with gastric cancer [106]. By authors, two shRNA (short hairpin RNAs) were administered against TRPM2 to lower the expression and role in gastric cancer, AGS, and MKN45 [109]. Outcomes proved that TRPM2 is practically expressed like a plasma membrane ion channel which is penetrable to Ca^2+^ in gastric cancer cells along with its impediment lowered cell bioenergetics, inhibited cell invasion, and declined cell survival. Further, these consequences were established in vivo through a SCID mouse model, and the decline of TRPM2 was directed to a decreased growth of tumor. The authors recognized that to facilitate gastric cancer survival TRPM2 functions through JNK-dependent as well as mTOR-independent pathways of autophagy [25, 109]. Later, Almasi et al. stated that the efficacy of chemotherapy drugs, paclitaxel and doxorubicin, is increased by the inhibition of TRPM2 which proposes that inhibition of TRPM2 in combination with established chemotherapeutics might be an effective approach to treat gastric cancer [109]. In addition, the authors stated that there is a correlation with the lower overall survival rate of patients particularly in late/advanced phases thus demonstrating the probability of possible function of TRPM2 as a prognostic biomarker for the late phase of gastric cancer [38, 109]. Likewise, further investigations have reported that elevated expression of TRPM5 was related to lower survival in patients suffering from gastric cancer [110]. However, additional research are required to confirm the significance of TRPM2 and TRPM5 in the survival and clinical outcomes of patients with gastric cancer.

### TRPM2 in prostate cancer

TRPM2 plays important role in the proliferation of prostate cancer cells [38, 111]. After depletion of TRPM2 with siRNA, the progress of prostate cancer, without non-cancerous cells, was lowered. Within non-cancerous cells, TRPM2 was located in the plasma membrane as well as in the cytoplasm, without in the nucleus. However, a major quantity of TRPM2 was located in the nucleus and the non-nuclear fraction of prostate cancer cells. The role of TRPM2 in the nucleus of cancer cells is unknown. These outcomes recommend that the reduced TRPM2 can be a therapeutic way to regulate prostate cancer development.

### TRPM2 in leukemia

In Jurkat cells firmly explicitly empty vector or Bcl-2, TRPM2 inhibition with N-(p- amylcinnamoyl) anthranilic acid (ACA) subsequently irradiation reduced phosphorylation of CAMKII along with closed radiation-prompted phosphorylation-dependent deactivation of cdc2 [38, 102]. Altogether ACA and clotrimazole, the nonspecific TRPM2 inhibitors, elevated cell death. Besides, TRPM2 breakdown considerably reduced the number of cells blocked in G2/M and declined viability. This finding proposes that irradiation excites Ca^2+^ entrance by TRPM2 that is excessive in Bcl-2 up-regulated T Cell leukemia cells, also donates to deactivation of G2/M cell cycle arrest, cdc2, and cell survival. Also, TRPM2 impediment discharge cells from G2/M arrest, leading to cell death. Those results recommend that the inhibited TRPM2 can be a therapeutic attitude to raise sensitivity within T-cell leukemia towards radiation. The expression pattern of TRPM2 ion channels in various cell lines is shorted in Table 1.

**Table 1:** The expression pattern of TRPM2 ion channels in various cell lines

**Primary cells or Cell lines**	**References**
Primary rat hepatocytes	[112, 113]
Primary mouse hepatocytes	[112]
H4IIE rat liver cell lines	[43]
SH-SY5Y neuroblastoma cells	[114]
BxPC-3 pancreatic cells	[115]
MCF-7 human breast adenocarcinoma cells	[100, 101]
MDA-MB-231 human breast adenocarcinoma cells	[100]
AGS gastric cancer cells	[106]
MKN-45 gastric cancer cells	[106]
A549 lung cancer cell lines	[116, 117]
H1299 lung cancer cell lines	[116]
PC-3 prostate cancer cells	[111]
DU-145 prostate cancer cells	[111]
Jurkat E6.1 T cell leukemia cells	[102]
SCC9 oral squamous carcinoma cells	[105]
Y79 retinoblastoma cell lines	[118]
Weri-Rb1 retinoblastoma cell lines	[118]
mpkCCD_cl4_ mouse kidney cortical collecting duct cells	[119]

## Potential pharmacological inhibitors of TRPM2 channel

TRPM2 channel is potentially regulated through a variety of factors, such as [Ca^2+^]_cyt_, H_2_O_2,_ cADPR (Cyclic adenosine 5′-diphosphate ribose), NAADP (Nicotinic acid adenine dinucleotide 2’-phosphate), and extra- and intracellular pH of the cells [19, 92]. It has been suggested the activation of poly (ADPR) polymerase causes the exposure of TRPM2 channels through H_2_O_2_ [12]. Poly (ADPR) polymerase is a universally expressed enzyme which catalyzes the break of NAD^+^ into nicotinamide and ADPR. Besides, TRPM2 action is escalated at increased [Ca^2+^]_i_ [76]. The range of active pharmacological modulators of TRPM2 is limited to the TRPM2 channel. Some of the pharmacological inhibitors identified so far are not selective blockers. A small amount of TRPM2 channel blockers have been recognized along with seemed to be cell-specific. For example, N-(p-amylcinnomoyl) anthranilic acid (ACA), the IP3R (inositol 1,4,5-trisphosphate receptor) inhibitor 2-aminoethoxydiphenyl borate or PLC inhibitor flufenamic acid (FFA) did not block the ADPR-induced Ca^2+^ influx within hippocampal cells of the rat. However, ACA and FFA curbed the entrance of Ca^2+^ in rat preliminary striatal cells [120]. Either ADPR or H_2_O_2_ can gate the TRPM2 channels in neuronal cells. It suggests that the accurate connection between TRPM2 channel stimulation as well as cell death now remains undetermined [120]. Determination of the ion channel structures is important for understanding the mechanisms of gating, ion permeation, and selectivity [99]. Proper structural knowledge of the channel can also play an important role in the development of selective inhibitors. Recent studies suggest that flufenamic acid [121], clotrimazole, econazole [12], ACA [15, 122], 2-APB [99], antioxidants, glycohydrolase inhibitors, PARP inhibitors, chlorpromazine AMP, 8 Br ADPR can act as non-selective inhibitors of TRPM2 channel. It has been known that intracellular acidosis may inhibit TRPM2 (pKa 6.7) [66, 92, 112, 113]. To know the details of the effects of TRPM2 channel inhibitors in mammalian cells, readers are requested to some recent comprehensive reviews [66, 123, 124]. Moreover, as CD38 signals to TRPM2 via ADPR [125] and arouses Ca^2+^ influx through TRPM2, therefore, another possibility of pharmacologic inhibition of TRPM2 is the inhibition of receptor-mediated activation of CD38 [25].

## Conclusion and future perspectives

A lot of work has been done on the activation of TRPM2 channels by ROS for different cell types including neuronal cells, liver cells, pancreatic, and cardiac cells. Now it is well-known that TRPM2 shows a crucial function in Ca^2+^ entry into different cell types subjected to ischemic reperfusion injury. Inhibitors of Ca^2+^ entry through TRPM2 have been shown to prevent IR injury in case of heart, liver, brain and/or kidney. Because it has been recognized that ROS-initiated stimulation of TRPM2 takes part together with acute and chronic liver injury, considerable additional investigation is required to explain the mechanisms engaged as well as the situations under which pharmacological hindrance of TRPM2 can be a selective clinical approach to diminish ROS-initiated liver injury. In the case of cancers, the expression pattern of TRPM2 in various kinds of cancer suggests that TRPM2 can stimulate tumor survival. Inhibited TRPM2 has been known to increase cell death along with augmenting sensitivity to chemotherapeutic agents including doxorubicin in several malignancies with neuroblastoma [71, 98], gastric cancer [109], T cell leukemia [102], triple-negative breast cancer cell lines [101]. The prevalence of statistics in cancer models confirms the idea that TRPM2 expression as well as its role has an essential part in conserving the viability of cancer cells- which provides an important therapeutic opportunity. Further research on the roles of TRPM2 channels in cancer and the evaluation of the pharmacological inhibition of TRPM2 in cancers may conceivably lead to improved and selective treatment regimens in the near future.

## Data Availability

Not Applicable.
